# Peripheral nerve abnormality in HIV leprosy patients

**DOI:** 10.1371/journal.pntd.0006633

**Published:** 2018-07-18

**Authors:** Marilia Brasil Xavier, Mariana Garcia Borges do Nascimento, Keila de Nazare Madureira Batista, Danusa Neves Somensi, Fernando Octavio Machado Juca Neto, Thomaz Xavier Carneiro, Claudia Maria Castro Gomes, Carlos Eduardo Pereira Corbett

**Affiliations:** 1 Nucleo de Medicina Tropical, Universidade Federal do Para, Belem, Para, Brazil; 2 Centro de Ciencias Biologicas e da Saude, Universidade do Estado do Para, Belem, Para, Brazil; 3 Faculdade de Medicina FMUSP, Universidade de Sao Paulo, Sao Paulo, Sao Paulo, Brazil; 4 Faculdade de Fisioterapia, Universidade Federal do Para, Belem, Para, Brazil; University of Tennessee, UNITED STATES

## Abstract

**Background:**

The geographical overlap of HIV (human immunodeficiency virus) and leprosy infection has become increasingly frequent and worrying, bringing many clinical issues. Peripheral neuropathy is very frequent in leprosy because of the predilection of its etiologic agent by Schwann cells of the peripheral nervous system, and it also affects individuals with HIV as one of the most common neurological manifestations.

**Methodology/Principal findings:**

The present study compared a cohort of 63 patients diagnosed with leprosy and coinfected with HIV with a cohort of 64 patients with leprosy alone, who were followed at the outpatient clinic of the Nucleus of Tropical Medicine of the Federal University of Pará, Brazil. We observed that HIV-coinfected leprosy patients presented greater odds of overall peripheral nerve damage (nerve function impairment—NFI) than patients with leprosy alone. More sensitive damage was observed, especially in patients coinfected with multibacillary forms. Leprosy patients coinfected with HIV presented higher chances of motor damage with improvement over time using multidrug therapy (MDT) and highly active antiretroviral therapy (HAART), along with a greater extent of damage and occurrence of neuritis. The data suggest that in addition to patients presenting possible damage caused by leprosy, they also had a greater damage gradient attributable to HIV disease, but not related to HAART because most of these patients had been on the treatment for less than a year. Neuritis was treated with prednisone at doses recommended by the WHO, and coinfected patients had the highest rate of clinical improvement in the first 60 days.

**Conclusions/Significance:**

The clinical characteristics of the two diseases should be considered in leprosy patients coinfected with HIV for better diagnosis and treatment of peripheral neuropathy. We suggest that new simplified assessment tools that allow the evaluation of the NFI of these patients be developed for use in the service.

## Author summary

Coinfections of leprosy and HIV are a challenge for clinical management, particularly concerning peripheral neuropathy, which is common in leprosy and is also present in HIV infection. We conducted a clinical follow-up of leprosy patients coinfected with HIV and compared them to patients with leprosy alone and observed that coinfected patients were more likely to present overall peripheral nerve damage (nerve function impairment—NFI), especially in multibacillary patients, where motor damage was largely responsible for this impairment. Throughout the period using multidrug therapy (MDT) and highly active antiretroviral therapy (HAART), the development of the damage, along with the occurrence of neuritis and complaints, decreased considerably. Thus, the data suggest that in addition to patients presenting possible damage caused by leprosy, they also have a greater damage gradient attributable to the disease due to HIV but not related to HAART. Another important point was the therapeutics used for neuritis with prednisone at doses recommended by the WHO, in which the coinfected patients had the highest rate of clinical improvement in the first 60 days. The characteristics of the two diseases should be considered in the understanding and clinical management of peripheral nerve alterations in leprosy patients coinfected with HIV. In addition, it became clear that further NFI assessment tests should be used with these patients.

## Introduction

Leprosy and human immunodeficiency virus (HIV) infection are two diseases of public health importance in the world. The increasing geographical overlap of these endemic and pandemic diseases may increase the number of individuals with both diseases in the world. In 2016, Brazil had an overall new case detection rate in leprosy of 12.2/100,000 inhabitants, and Pará had an overall new case detection rate of 40.4/100,000 inhabitants, which is considered hyperendemic [1].

The acquired immunodeficiency syndrome (AIDS) caused by HIV is an extraordinary type of public health crisis; it is both an emergency and a long-term problem. In 2016, 37,884 cases of the infection were reported, with an incidence rate of syndrome of 18.5 cases per 100,000 inhabitants. In 2016, Pará had the 4th highest detection rate among Brazilian states, and its capital, Belém, had the 3rd highest detection rate (55.4/100,000 inhabitants) among Brazilian capitals [2]. The geographical overlap of the two diseases has become increasingly present and worrisome, which raises questions about how the health professionals address the clinical management and therapeutics of an individual coinfected by both diseases [3–6].

Leprosy is considered a disease of the skin and peripheral nerve trunks [7]. Neural lesions are the result of changes in the sensory, motor and autonomic systems. Leprosy is a spectral disease, ranging from tuberculoid leprosy, in which the host has good cell-mediated immunity against *M*. *leprae* with limited disease, to the lepromatous form, in which the host is anergic to *M*. *leprae* and the organism proliferates in the skin, peripheral nerves and other systems. At the center of this spectrum are intermediate forms called borderlines [8,9].

At the beginning of the HIV epidemic, it was anticipated that this infection would lead patients to develop the most severe form of leprosy. However, this has not occurred, and studies have begun to note that HIV/leprosy coinfection seems to be directly related to the immune improvement provided by the introduction of antiretroviral therapy (HAART) and therefore, leading to the development of the milder and limited form of the disease [10–15].

Some studies report a higher prevalence of tuberculoid (TT) and borderline-tuberculoid (BT) forms and a higher occurrence of reactions and neuritis in coinfected patients [4–5,16]. Therefore, these patients may potentially have a greater risk of prolonged reactions and neural damage, also due to other neurotropic infections and to the toxicity of antiretroviral drugs and in several treatments of comorbidities [17,18].

In the case of *M*. *leprae* infection, several mechanisms can trigger nervous aggression, depending on the clinical forms and the different periods of the disease. They are essentially established in two ways: an early phase, which occurs without inflammation, likely by the action of the bacillus in the nerves, and a late phase, in which there is an inflammatory process resulting from the action of the body in response to the presence of the bacillus [19–22].

Bacillary invasion and, especially, the inflammatory process of the peripheral nerves cause sensory-motor damage, which generates symptoms of paresthesia and hyperalgesia, although in some cases, the alterations in pain, thermal and tactile sensitivity occur quietly [23].

Neurological changes associated with HIV infection have decreased with the use of HAART. However, some complications continue to have a serious impact on the lives of these patients [17]. Peripheral neuropathy is very common in leprosy because of the predilection of its etiologic agent for Schwann cells of the peripheral nervous system; it also affects individuals with HIV/AIDS at a frequency ranging from 30% to 50%, which makes it one of the most common neurological manifestations in these patients. The neuropathy can occur at any stage of HIV infection, and with the passage of time and prolonged use of HAART, peripheral neuropathies tend to be more frequent [17,18,24].

Corticosteroids are among the drugs of choice for the treatment of leprosy neuropathy and are widely used to treat acute nerve damage. These drugs have been used to treat inflammation associated with leprosy neuropathy since the 1970s. Unfortunately, however, follow-up studies are scarce, and coinfected patients are very rare [25].

Investigations on the clinical and therapeutic aspects of HIV and leprosy coinfection with emphasis on neural damage are valuable tools for the management of this type of patient and are also important for preventive action to combat the incapacities that are the consequences of nerve involvement. Thus, this study aimed to describe the clinical and therapeutic aspects of neurological alterations, such as sensory and motor damage, neuritis, neuropathic pain and complaints related to neural damage in patients coinfected with HIV and leprosy.

## Methods

### Study design

This is a comparative study of two historical clinical cohorts of patients diagnosed with leprosy and seen at the dermatology outpatient clinic of the Tropical Medicine Nucleus of the Federal University of Pará (NMT/UFPA), located in the city of Belém, capital of the state of Pará, Brazil. The first cohort was composed of 63 leprosy patients coinfected with HIV, and the second cohort was composed of 64 leprosy patients who were not HIV coinfected from 2007 to 2017 Patient follow-up was performed on average up to 12 months for paucibacillary and 24 months for multibacillary leprosy patients, even after stopping multidrug therapy (MDT).

### Inclusion criteria

We included patients from 18 to 70 years of age diagnosed with leprosy and treated with MDT as recommended by the Ministry of Health [26], classified according to Ridley and Jopling [8], and diagnosed with HIV/AIDS according to the Brazilian Ministry of Health [27].

### Study procedures

#### Outcomes definitions

Neuritis: nerve damage with altered sensory and/or motor function with variable pain symptomatology; Acute neuritis: intense pain occurring spontaneously or during palpation of the nerve trunks, with evolution up to 3 months; Chronic neuritis: nerve damage of insidious onset and slow progression with variable pain symptomatology and over 3 months; Silent neuritis: alteration of sensory and/or motor function in the absence of pain; Neuropathic pain: presence of symptoms (pain, allodynia, hyperpathy, paresthesias) without progressive loss of neural, sensory and motor function; Sensitive damage: result of the sensitivity test with Semmes Weinstein monofilaments exceeding 2 g; Motor damage: Voluntary Muscle Test result equal to or less than 4; Nerve Function Impairment (NFI): nerves that presented some alteration of sensory and/or motor function; Clinical complaint related to sensory damage: pain or paresthesias (numbness, tingling, needling, shock and burning); Clinical improvement of neuritis: clinical improvement in four weeks with corticoid use.

#### Patients and methods

The patients were submitted to dermatoneurological examination by dermatologists, infectologists, neurologists, nurses and physical therapists. HIV and AIDS infection and the use of HAART were defined according to the guidelines of the Brazilian Ministry of Health with positive serology ELISA and western blot, flow cytometry for peripheral blood CD4 counts and criteria for AIDS (defined as a CD4 T lymphocyte count of 200 cells/mL and/or clinical conditions that define the disease) (MS, 2017). The diagnosis and classification of leprosy were made according to clinical criteria [28], complementary bacilloscopy and histopathology exams [9] and the WHO Operational Classification [29].

NFI considered the sensory damage that was assessed by sensitivity tests on the nerves most commonly affected in leprosy (ulnar, median, radial, common fibular and posterior tibial), performed using Semmes Weinstein monofilament, in which individuals with sensory damage were considered those who did not feel the 2-g filaments. Motor damage was evaluated by the manual muscle strength test, which is also related to the nerves present in the evaluation, and motor damage was considered when the patient presented a degree of muscle strength of less than or equal to 4 (paralyzed or weak) [28]

Patients with paucibacillary leprosy (PB) patients, that means TT and BT forms, were treated for 6 months with rifampicin and dapsone, and multibacillary leprosy (MB) patients—Borderline-Borderline (BB), Borderline-Lepromatous (BL) and Lepromatous-Lepromatous (LL) forms—were treated for 12–24 months with rifampicin, clofazimine and dapsone. Primary neural patients (PNL) were classified as PB or MB according to the number of affected nerve trunks. In case of neuritis, steroids were administered at the dose of 1–2 mg per kg of body weight per day and were slowly withdrawn according to clinical parameters [28]

#### Statistical analysis

The Statistical Package for Social Sciences (SPSS), version 22.0, was used, with the Mann-Whitney test for comparison of medians, the chi-square test and the G-test when pertinent, along with calculations of odds ratios and relative risk. The significance level was 0.05, and the confidence interval was 95%. The missing data were taken from the calculation.

#### Ethics statement

The norms for research in human established by Resolution 466/12 of the National Health Council were followed, as were the recommendations of the ethics committee in human research at the University of São Paulo (USP), with approval granted in accordance with Opinion No. 2.060.860. All study participants provided written informed consent in agreeing to participate in the research. This study follows the STROBE (STrengthening the Reporting of OBservational studies in Epidemiology) guidelines and the STROBE checklist is included (S1 Checklist).

## Results

The study included a sample of 63 HIV/leprosy-coinfected patients and 64 HIV-free leprosy patients. Men (n = 80, 63.0%) with a mean age of 40 years were the most affected in both groups. Paucibacillary forms were more frequent in the coinfected patients, and multibacillary forms were more frequent in the non-coinfected patients. The LL clinical form and the primary neural form were not observed in coinfected patients (Table 1).

**Table 1:** Distribution of coinfected and non-coinfected patients according to demographical and clinical variables.

Variables	No. observations (%) or Mean ± SD			Statistical test (95% CI) ^+^ *
	Coinfected	Non-coinfected	Total	
**Observations (n = 127)**	63 (49.6)	64 (50.4)	127 (100)	
**Gender**				
Male	45 (71.3)	35 (54.7)	80 (63.0)	Chi-square, p > 0.05
Female	18 (28.7)	29 (45.3)	47 (37.0)	
**Age (years)** ^a^	39.7 ± 9.6	40.6 ± 14.8	40.2 ± 12.5	0.87 (-3.51–5.26)
**Clinical Form**				
TT	17 (27.0)	16 (25.0)	33 (26.0)	
BT	20 (31.8)	12 (18.8)	32 (25.2)	
BB	20 (31.8)	24 (37.5)	44 (34.7)	
BL	6 (9.5)	5 (7.8)	11 (8.7)	G-test, p > 0.05 ^b^
LL	0 (0.0)	2 (3.1)	2 (1.6)	
PNL	0 (0.0)	5 (7.8)	5 (3.9)	
**Operational Classification**				
Paucibacillary (TT, BT, PNL)	37 (58.7)	29 (45.3)	63 (49.6)	Chi-square, p > 0.05
Multibacillary (BB, BL, LL, PNL)	26 (41.3)	35 (54.7)	64 (50.4)	

^+^ Exact p-values for estimated comparison reported only when statistically significance was achieved; non-significance indicated through confidence intervals (95% CI) containing their null values;

* Non-coinfected patients used as comparison group (baseline);

^a^ Mean difference with Two-sample t test using Welch’s approximation.

^b^ Sample comprised by all clinical forms except neural and indeterminate, totalising 120 patients.

In the cohort of coinfected patients, most were using HAART (n = 55, 87.3%); only 8 (12.7%) patients were not using the therapy. The most frequent onset of leprosy in relation to HAART was within 12 months of the introduction of therapy. Only 13 (26%) patients presented leprosy after 12 months of HAART (S1 Table).

Coinfected patients presented a slightly higher chance of developing sensory damage but without significant differences. However, when considering bacillary disease, multibacillary coinfected patients were more likely to evolve with sensitive damage than paucibacillary coinfected patients. The motor damage was very obvious in coinfected patients diagnosed until the end of MDT, and a decrease was observed at that time. This fact is also observed in the extent of this damage, which was significantly higher in the coinfected patients (Table 2).

**Table 2:** Logistic regression for Adjusted Odds Ratios (OR) of occurrence of sensitive and motor neural damage and modified poisson regression for Adjusted Rate Ratio (RR) of sensitive and motor nerve damage rate.

Predictors variables	OR, (95% CI), p-value				Nerve Damage Rate ^a^ RR (95% CI), p-value
	All Phases ^b^ (n = 375)	Enrollment (n = 127)	During MDT (n = 127)	Discharge of MDT (n = 127)	All Phases ^b^ (n = 381)
	**Occurrence of Sensitive Nerve Damage**				
**Groups**					
Leprosy	1 [Reference]	1 [Reference]	1 [Reference]	1 [Reference]	1 [Reference]
Coinfected	1.44 (0.9–2.32), >.05	1.69 (0.74–3.83), >.05	1.86 (0.81–4.3), >.05	0.95 (0.41–2.2), >.05	1.11 (0.9–1.38), >.05
**WHO Classification**					
Paucibacillary (TT, BT, PNL)	1 [Reference]	1 [Reference]	1 [Reference]	1 [Reference]	1 [Reference]
Multibacillary (BB, BL, LL, PNL)	4.3 (1.2–15.37), .025	2.95 (0.34–25.72), >.05	6.52 (0.58–72.9), >.05	5.65 (0.5–64.47), >.05	3.48 (1.96–6.17), < .001
	**Occurrence of Motor Nerve Damage**				
**Groups**					
Leprosy	1 [Reference]	1 [Reference]	1 [Reference]	1 [Reference]	1 [Reference]
Coinfected	3.07 (1.8–5.23), < .001	7.05 (2.21–22.52), .001	3.29 (1.33–8.13), .01	1.68 (0.71–4), >.05	2.01 (1.5–2.71), < .001
**WHO Classification**					
Paucibacillary (TT, BT, PNL)	1 [Reference]	1 [Reference]	1 [Reference]	1 [Reference]	1 [Reference]
Multibacillary (BB, BL, LL, PNL)	2.02 (0.58–6.99), >.05	1.62 (0.19–13.69), >.05	2.03 (0.22–18.43), >.05	2.51 (0.29–21.5), >.05	1.55 (0.81–2.95), >.05

^a^ Patients Nerve Damage Rate calculated as the number of nerves with sensitive and/or motor damage (NFI) by the total of nerves examined (10 nerves per patient);

^b^ All phase analysis Adjusted by study phase

Considering the clinical forms of the leprosy spectrum according to Ridley Jopling and according to WHO, a greater risk of evolving with any neural damage was not observed; however, for patients of the same bacillary and clinical forms in both groups, coinfected patients experienced 18–61% more extensive neural impairment (Table 3).

**Table 3:** Logistic regression for Adjusted Odds Ratios (OR) of occurrence of any^a^ nerve function impairment and modified poisson regression for Adjusted Rate Ratio (RR) of nerve damage rate.

Predictors variables	Occurrence of NFI				Nerve Damage Rate ^b^
	OR, (95% CI), p-value				RR (95% CI), p-value
	All Phases ^c^ (n = 375)	Enrollment (n = 125)	During MDT (n = 125)	Discharge of MDT (n = 125)	All Phases ^b^ (n = 381)
**Groups**					
Leprosy	1 [Reference]	1 [Reference]	1 [Reference]	1 [Reference]	1 [Reference]
Coinfected	1.52 (0.97–2.38), >.05	2.33 (1.06–5.11), .034	1.75 (0.79–3.91), >.05	0.87 (0.4–1.91), >.05	1.41 (1.18–1.68), < .001
**WHO Classification**					
Paucibacillary (TT, BT, PNL)	1 [Reference]	1 [Reference]	1 [Reference]	1 [Reference]	1 [Reference]
Multibacillary (BB, BL, LL, PNL)	2.0 (0.56–7.1), >.05	1.35 (0.16–11.21), >.05	2.38 (0.19–30.09), >.05	2.44 (0.29–20.71), >.05	2.27 (1.46–3.51), < .001
**Clinical Presentation**					
TT	1 [Reference]	1 [Reference]	1 [Reference]	1 [Reference]	1 [Reference]
BT	1.2 (0.63–2.27), >.05	1.24 (0.41–3.73), >.05	1.5 (0.5–4.55), >.05	0.93 (0.29–2.91), >.05	1.11 (0.81–1.52), >.05
BB	1.74 (0.43–7.06), >.05	2.48 (0.24–26.09), >.05	2.22 (0.14–34.66), >.05	1.04 (0.1–10.96), >.05	1.18 (0.71–1.96), >.05
BL	2.04 (0.44–9.38), >.05	2.08 (0.16–27.1), >.05	1.72 (0.09–32.32), >.05	2.57 (0.19–35.64), >.05	1.07 (0.62–1.86), >.05
LL	1 [empty]	1 [empty]	1 [empty]	1 [empty]	2.87 (1.52–5.43), .001
PNL	2.91 (0.57–14.94), >.05	4.35 (0.31–61.57), >.05	3.96 (0.11–138.13), >.05	1.61 (0.11–22.97), >.05	1.48 (0.82–2.68), >.05

^a^ Any neural damage positive events defined as the occurrence of either sensitive or motor nerve damage;

^b^ Patients Nerve Damage Rate calculated as the number of nerves with motor, sensitive or both motor and sensitive damage by the total of nerves examined (10 nerves per patient);

^c^ All phase analysis Adjusted by study phase

Coinfected patients had a higher prevalence of neuritis (33.3%) at the time of diagnosis than those with leprosy alone, regardless of the clinical form (WHO) (17.2%). Coinfected patients appeared to evolve better from acute and chronic neuritis and remain with silent neuritis, differently from non-coinfected patients. Acute neuritis was more prevalent in coinfected patients at the beginning of treatment, with 9.5% (n = 6) versus 4.7% among non-coinfected patients (n = 3) (S2 Table).

Neuropathic pain occurred in a multibacillary form patient in each group at the end of treatment. These observations may have been impaired by the number of patients.

Considering NFI, the average number of affected nerve trunks was significantly higher in the coinfected group at the time of diagnosis, but throughout the treatment and at the end of MDT, this difference was not observed (Table 4).

**Table 4:** Distribution of coinfected and non-coinfected patients according the mean of NFI at all the moments of study.

Variables	No. Observation (%), Mean ± SD					Statistical test
	Coinfected		Non-coinfected		Total	
	PB	MB	PB	MB		
**Observations (n = 127)**	37 (58.7)	26 (41.3)	29 (45.3)	35 (54.7)	127 (100)	
**Enrollment**	0.97 ± 1.21	2.48 ± 2.79	0.68 ± 1.94	2.00 ± 2.27	1.53 ± 2.21	Mann-Whitney^1^, p = 0.0355
**During MDT**	0.52 ± 1.35	1.75 ± 2.47	0.65 ± 1.81	2.22 ± 1.94	1.30 ± 2.03	Mann-Whitney^2^, p>0.05
**Discharge of MDT**	0.58 ± 1.15	1.96 ± 2.62	0.44 ± 0.86	2.05 ± 2.07	1,27 ± 1,94	Mann-Whitney^3^, p>0.05

^1^ intergroup analysis of paucibacillary coinfected and non-coinfected at the time of enrollment;

^2^ intergroups analysis for the mean of NFI at during MDT to paucibacillary and multibacillary patients;

^3^ intergroup analysis for the mean of NFI at discharge of MDT to paucibacillary and multibacillary patients.

Regarding the complaints related to sensory impairment, the prevalence of sensory complaints was slightly higher in the coinfected than in the non-coinfected group, with significant improvement in this group over time, different from patients with leprosy alone. Follow-up losses may have impaired the analysis (S3 Table).

**Fig 1:**
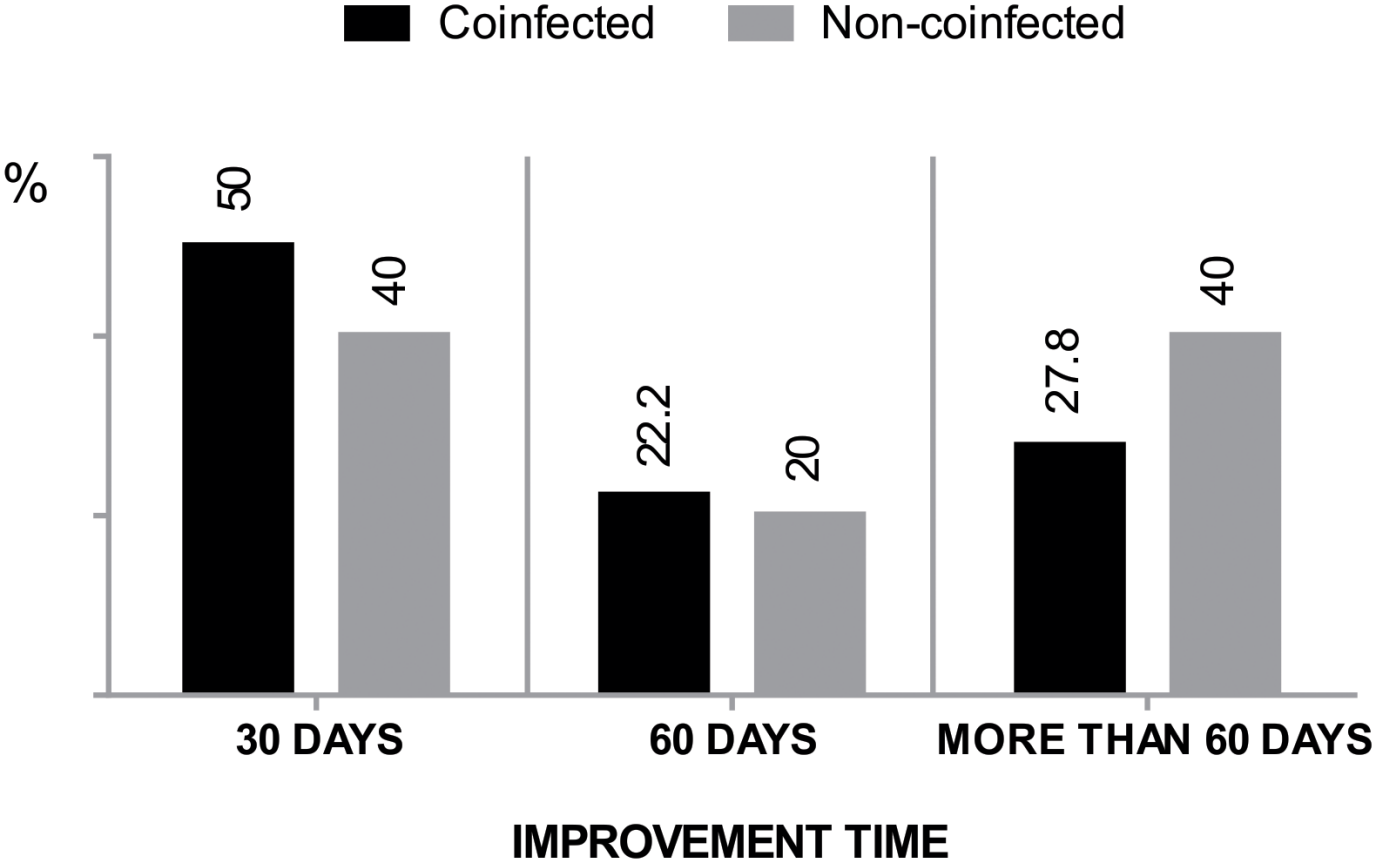
Time of neuritis improvement after use of drug therapy in coinfected and non-coinfected patients. * G-test, p>0.05.

The use of corticosteroids alone was the regimen most used in the treatment of neuritis, followed by corticosteroids and amitriptyline and corticosteroids and paracetamol. In the comparison of the clinical improvement outcome in patients from each cohort who used corticosteroids alone or in association with an analgesic and/or antidepressant, a good response was observed in the coinfected patient group, with a higher frequency of patients with faster regression, in the three moments of observation, but without a significant difference in relation to non-coinfected patients (Fig 1).

## Discussion

Peripheral nerve changes are the main cause of disability, pain and paresthesias and have an impact on the quality of life of leprosy patients [30–32]. Inflammatory reactive states of leprosy are the main cause of nerve changes in these patients, but there are other pathological mechanisms capable of causing such alterations [4–5, 30,33].

HIV infection has a large number of neurological manifestations that occur in the amplitude of the immune spectrum of the disease and in the course of the disease [17, 33–36].

In this study, a cohort of coinfected leprosy patients was observed using simplified evaluation tests, widely validated for the evaluation of sensory and motor peripheral damage in leprosy. The great question arising from the empirical reality and whose answer is fundamental to the management of the patient is to know how this nerve damage behaves in patients who have HIV in addition to leprosy. With the interaction between these endemic and pandemic disease, the complexity of the interfering factors is a challenge for researchers [3, 30, 37–38].

Our demographic results demonstrate agreement with the literature and also indicate that leprosy/HIV is the most common coinfection in men of young adult age with an average of 40 years [37]. Paucibacillary forms were more frequent in the coinfected group, and multibacillary forms were more frequent in the non-coinfected group, confirming what has been reported in the literature [4,5,13,39–41]. In fact, the diagnosis of leprosy in patients with HIV infection has been associated with the improvement of patient immunity after the initiation of HAART and was characterized by elevated CD4 ^+^ T cells in these patients [11–12, 42–44].

Overall nerve function impairment (NFI) was significantly higher in coinfected patients. Both the sensory damage and motor damage usually occurred more often in patients who, in addition to having possible damage due to leprosy, also had a greater gradient attributable to HIV disease. Thus, the clinical characteristics of the two diseases should be considered to better understand this damage [30–31,33,45–46].

Other studies in our group, using other tools and some instigating data, point to the need for simplified but more specific tools for the management of coinfected patients, especially in areas where diseases occur endemically [1–2,6], whose data are available and not yet published [45,46].

The mechanisms of peripheral damage widely studied in leprosy are still difficult to understand, diagnose and treat [30,33,36,47]. The university outpatient clinic where the research was conducted has been a reference clinic for the treatment of leprosy for over 20 years and is currently also a reference to the coinfected patients. Thus, the approaches to the diagnosis of damage and patient management are made from the perspective of the knowledge of the peripheral nerve damage of leprosy patients, in the same way that other authors have described in the literature [31, 45–46].

The neuropathies associated with HIV disease described in the literature include Symmetric Distal Sensorimotor Polyneuropathy, which is the most common form and occurs mainly in patients who do not use HAART and in later stages of the disease [48–49]. Toxic neuropathies occur due to mitochondrial changes and occur in patients taking HAART [48]. Progressive polyradiculopathies have also been described in patients before the HAART era and currently occur less frequently and in the later phase of the disease. The condition is directly related to cytomegalovirus infection and CD4 ^+^ lymphocyte counts below 200 cells/mm^3^ [48,50]. Demyelinating inflammatory polyneuropathies may occur in the early stages of the disease in asymptomatic patients and may occur acutely or chronically, the latter being the most common [48, 50].

The patients described herein were mostly using HAART (87%), and more than half of these had used HAART for less than one year. Considering that these patients and those who were not using HAART at the diagnosis of leprosy presented sensory and motor alterations, the damage does not seem attributable to the neurotoxicity of antiretroviral drugs, leading us to believe that NFI could be the sum of the sensory and motor damage caused by leprosy plus the motor sensory damage caused by HIV described in the literature in patients who do not use HAART [48,49]. This type of HIV neuropathy tends to improve with the use of HAART, which could explain the improvement in NFI during the moments of study observation, which, although they had been guided by MDT, also reflect the use of HAART over time.

We know that AIDS is a disease that has comorbidities, with overall weight loss being a criterion for defining the disease. Therefore, these patients who have been diagnosed with AIDS could have repercussions in the evaluation of certain factors, such as muscle strength. The same may occur in later stages of the disease in patients taking antiretroviral drugs where neurotoxicity and the impact of drugs on muscle loss (lean mass) could also affect muscle strength. However, the loss of lean mass occurs much later, after approximately 90 months of HAART use [51–53].

However, greater motor damage was evident in patients coinfected at the time of diagnosis until stopping MDT, decreasing at the end, and in this case regardless of bacillary activity. The months of observation of MDT are also months in which the patient was using HAART, which means an overall improvement in the patient’s condition and certainly of afflictions that could have been causing repercussions as both sensory and motor damage [45–46, 54].

Leprosy/HIV coinfection seems to be directly related to the immune improvement that the introduction of HAART provides due to a phenomenon called immune reconstruction inflammatory syndrome (IRIS), which constitutes the immune response to a specific pathogen after the introduction of antiretroviral therapy [10–12, 14–15, 43, 55]. The change in immunity caused by the immune restoration also provides the patient’s inflammatory response to the nerve trunks and nerve endings of the skin. The inflammatory mechanism of neural damage in leprosy is most evident at the time neuritis occurs. However, if the patient has a greater chance of neuritis attributable also to leprosy, with repercussions on NFI due to the restoration of the immune response, HAART also restores or contributes to remodeling patient immune response or immune memory, which may justify the better evolution of patients observed in this study, evolving with a lower prevalence of neuritis at the end of MDT treatment when compared with non-coinfected patients [4, 12].

The odds of the occurrence of damage and the extent of damage were higher in coinfected individuals and those who apparently showed a more rapid improvement at the moments observed, which could be related to the improvement of overt neuritis, which manifest pain. It is noteworthy that AIDS alters the overall immunity of the patient; after immune reconstitution, the specific response will be re-established [54].

In response to abrupt episodes of bacillary proliferation or rapid variations in cellular immunity, rapid formations of granulomas with macrophages, epithelioid and lymphocyte cells, edema and, in some cases, necrotic changes occur. These changes lead to the worsening of neurological lesions, which manifest themselves through complaints such as paresthesias, pain and motor and sensory deficits and reflect higher degrees of disability [56].

There was no difference in the frequency of complaints, but it is noteworthy that there were slightly more complaints among coinfected patients at the time of the leprosy diagnosis than among non-coinfected patients, who seemed to present a more rapid improvement, which can be attributed to the fact that when coinfected patients initiate treatment for leprosy, they generally are more affected by reactions and neuritis as a result of the immunological changes [10, 13–15, 43]. However, attention must be paid to the subjective character of pain because the complaints were present among individuals who did not present neuritis and even in individuals without sensory and motor damage. The complaints may have other non-neuropathic origins, including psychological ones, because of the high stigma surrounding both diseases [57].

To prevent nerve damage from being permanent, it is essential that it is treated early. Prednisolone is a potent immunosuppressive recommended for the treatment of patients with leprosy. However, effective optimal doses and treatment times for controlling the damage are still unknown. Studies in coinfected patients are still scarce [4, 25, 58]. The observation of the best clinical improvement rate in coinfected patients using steroids, especially in the first 60 days, points to a possible factor unrelated only to the leprous neuritis.

Based on these results, we can assume that the corticosteroid is effective due to the improvement of factors related to HIV disease in coinfected patients. Patients with leprosy alone demonstrated much slower clinical improvement. A long-term cohort clinical trial demonstrated that improvements to leprosy damage occur in the first few weeks of corticosteroid use, while even high doses do little to contribute to chronic and persistent neuritis [59]. These intriguing aspects that require other studies for such observations to be corroborated. The number of patients was a limiting factor for this observation.

In the analysis of data obtained by clinical observation with simplified techniques, validated for the observation of leprosy neural damage in the two cohorts, and with the points considered in this discussion, it is strongly suggested that the greatest motor damage of the coinfected patient is likely not due to damage caused by *M*. *leprae* but rather to loss of body mass and other conditions associated with HIV disease, even those of central nervous origin [36]. In addition, it is strongly suggestive that the sensory damage can be attributed to the damages caused by both *M*. *leprae* and HIV. Therefore, further research is necessary, with specific complementary tests evaluating the nerve trunks of coinfected patients, studies on histopathological and immune and inflammatory responses of peripheral nerves, electroneuromyography studies and specific tests of muscular strength and the inclusion of a larger number of clinical variables of HIV disease.

We can also note that new simplified evaluation instruments that allow the evaluation of the NFI of leprosy patients coinfected with HIV should be validated for use in the service because such diseases occur in areas where such comorbidities are still considered neglected diseases and affect an economically active and socially vulnerable population.

## Supporting information

S1 Table Distribution of coinfected patients according to clinical variables. ^a^ intragroup analysis of paucibacillary and multibacillary coinfected.
(PDF)

S2 Table Distribution of coinfected and non-coinfected patients according to occurrence of neuritis. ^a^ intragroup analysis of paucibacillary and multibacillary coinfected; ^b^ intragroup analysis of paucibacillary and multibacillary non-coinfected; ^1^ intergroup analysis of paucibacillary of coinfected and non-coinfected group; ^2^ intergroup analysis of multibacillary of coinfected and non-coinfected group.
(PDF)

S3 Table Distribution of coinfected and non-coinfected patients according to complaints related to sensitive neural damage. ^a^ without information coinfected = 1, without information non-coinfected = 0; ^b^ Without information coinfected = 18, without information non-coinfected = 14; ^c^ without information coinfected = 5, without information non-coinfected = 6; ^1^ intergroup analysis for paucibacillary and multibacillary at all three moments of the study.
(PDF)

S1 Checklist STROBE checklist. (PDF)
