# Supplementary material for: Peripheral nerve abnormality in HIV leprosy patients
Source: PLoS Negl Trop Dis. 2018 Jul 18;12(7):e0006633. doi: 10.1371/journal.pntd.0006633 (PMC6066254; doi:10.1371/journal.pntd.0006633)
Supplement: S1 Table — a intragroup analysis of paucibacillary and multibacillary coinfected. (PDF) [file pntd.0006633.s001.pdf]

| Variables | No. Observation (%) | | | Statistical test |
|---|---|---|---|---|
| | Coinfected [a] | | Total | |
| | PB | MB | | |
| **Observations (n=63)** | 37 (58.7) | 26 (41.3) | 63 (100) | Chi-square [a], p>0.05 |
| **HAART** | | | | |
| With HAART | 32 (86.5) | 23 (88.5) | 55 (87.3) | G-Test [a,] p>0.05 |
| Without HAART | 5 (13.5) | 3 (11.5) | 8 (12.7) | |
| **Time of HAART vs. diagnosis of leprosy** | | | | |
| Before HAART | 11 (29.7) | 8 (30.8) | 19 (30.2) | |
| 0 - 6 months | 14 (37.8) | 7 (26.9) | 21 (33.3) | G-Test [a,] p>0.05 |
| >6 - 12 months | 8 (21.6) | 2 (7.7) | 10 (15.9) | |
| More than 1 year | 5 (13.5) | 8 (30.8) | 13 (20.6) | |
