# Supplementary material for: Peripheral nerve abnormality in HIV leprosy patients
Source: PLoS Negl Trop Dis. 2018 Jul 18;12(7):e0006633. doi: 10.1371/journal.pntd.0006633 (PMC6066254; doi:10.1371/journal.pntd.0006633)
Supplement: S2 Table — a intragroup analysis of paucibacillary and multibacillary coinfected; b intragroup analysis of paucibacillary and multibacillary non-coinfected; 1 intergroup analysis of paucibacillary of coinfected and non-coinfected group; 2 intergroup analysis of multibacillary of coinfected and non-coinfected group. (PDF) [file pntd.0006633.s002.pdf]

Variables	No. Observation (%)						Statistical test
	Coinfected [a]			Non-coinfected [b]			
	PB	MB	Total	PB	MB	Total	
**Observations (n=127)**	37 (58.7)	26 (41.3)	63 (100)	29 (45.3)	35 (54.7)	64 (100)	
**Enrollment**							
Acute neuritis	2 (5.4)	4 (15.4)	6 (9.5)	1 (4.3)	2 (5.7)	3 (4.7)	G-Test [a], p=0.0151
Chronic neuritis	1 (2.7)	6 (23.1)	7 (11.1)	0 (0.0)	5 (14.3)	5 (7.8)	G-Test [b], p=0.0220
Silent neuritis	7 (18.9)	1 (3.8)	8 (12.7)	3 (10.3)	0 (0.0)	3 (4.7)	G-Test [1, 2], p>0.05
Without neuritis	27 (73.0)	15 (57.7)	42 (66.6)	25 (86.2)	28 (80.0)	53 (82.8)	
**During MDT**							
Acute neuritis	1 (2.7)	2 (7.7)	3 (4.8)	0 (0.0)	7 (20.0)	7 (10.9)	G-Test [a], p > 0.05
Chronic neuritis	1 (2.7)	4 (15.4)	5 (7.9)	1 (3.4)	4 (11.4)	5 (7.8)	G-Test [b], p=0.0068
Silent neuritis	6 (16.2)	4 (15.4)	10 (15.9)	2 (6.9)	0 (0.0)	2 (3.1)	G-Test [1], p>0.05
Without neuritis	29 (78.4)	16 (61.5)	45 (71.4)	26 (89.7)	24 (68.6)	50 (78.1)	G-Test [2], p=0.0444
**Discharge of MDT**							
Acute neuritis	0 (0.0)	0 (0.0)	0 (0.0)	0 (0.0)	1 (2.9)	1 (1.6)	
Chronic neuritis	0 (0.0)	1 (3.8)	1 (1.6)	0 (0.0)	2 (5.7)	2 (3.1)	G-Test [a, b], p > 0.05
Silent neuritis	8 (21.6)	1 (3.8)	9 (14.3)	2 (6.9)	0 (0.0)	2 (3.1)	G-Test [1, 2], p > 0.05
Without neuritis	29 (78.4)	24 (92.4)	53 (84.1)	27 (93.1)	32 (91.4)	59 (92.2)	
