# Supplementary material for: Peripheral nerve abnormality in HIV leprosy patients
Source: PLoS Negl Trop Dis. 2018 Jul 18;12(7):e0006633. doi: 10.1371/journal.pntd.0006633 (PMC6066254; doi:10.1371/journal.pntd.0006633)
Supplement: S3 Table — a without information coinfected = 1, without information non-coinfected = 0; b Without information coinfected = 18, without information non-coinfected = 14; c without information coinfected = 5, without information non-coinfected = 6; 1 intergroup analysis for paucibacillary and multibacillary at all three moments of the study. (PDF) [file pntd.0006633.s003.pdf]

Variables	No. Observation (%)						Statistical test
	Coinfected			Non-coinfected			
	PB	MB	Total	PB	MB	Total	
**Observations (n=127)**	37 (58.7)	26 (41.3)	63 (100)	29 (45.3)	35 (54.7)	64 (100)	
**Enrollment[a]**							
No complaints	17 (45.9)	4 (15.4)	21 (33.3)	17 (58.6)	5 (14.3)	22 (34.4)	
Pain	6 (16.2)	4 (15.4)	10 (15.9)	4 (13.8)	7 (20.0)	11 (17.2)	
Paresthesia	7 (18.9)	12 (46.2)	19 (30.2)	3 (10.3)	13 (37.1)	16 (25.0)	
Both pain and paresthesia	6 (16.2)	6 (23.1)	12 (19.0)	5 (17.2)	10 (28.6)	15 (23.4)	
**During MDT[b]**							
No complaints	15 (40.5)	6 (23.1)	21 (33.3)	11 (37.9)	9 (25.7)	20 (31.3)	
Pain	3 (8.1)	4 (15.4)	7 (11.1)	1 (3.4)	8 (22.9)	9 (14.1)	G-test,[1] $p>0.05$
Paresthesia	3 (8.1)	10 (38.5)	13 (20.6)	3 (10.3)	12 (34.3)	15 (23.4)	
Both pain and paresthesia	2 (5.4)	2 (7.7)	4 (6.3)	1 (3.4)	5 (14.3)	6 (9.4)	
**Discharge of MDT[c]**							
No complaints	23 (62.2)	15 (57.7)	38 (60.3)	14 (48.3)	10 (28.6)	24 (37.5)	
Pain	4 (10.8)	1 (3.8)	5 (7.9)	3 (10.3)	4 (11.4)	7 (10.9)	
Paresthesia	4 (10.8)	5 (19.2)	9 (14.3)	4 (13.8)	14 (40.0)	18 (28.1)	
Both pain and paresthesia	3 (8.1)	3 (11.5)	6 (9.5)	3 (10.3)	6 (17.1)	9 (14.1)	
